# The immune-cardiovascular metabolic circuitry in myocardial ischemia-reperfusion injury: from metabolic signal release to spatiotemporal reprogramming

**DOI:** 10.3389/fimmu.2026.1848067

**Published:** 2026-05-26

**Authors:** Conghao Tan, Junjie Zhou, Jiawen Li, Xinyi Zhang, Wei Yuan

**Affiliations:** 1 Department of Cardiology, Affiliated Hospital of Jiangsu University, Zhenjiang, China; 2 Institute of Cardiovascular Diseases, Jiangsu University, Zhenjiang, China

**Keywords:** immune cells, immunometabolism, metabolic reprogramming, myocardial ischemia-reperfusion injury, spatiotemporal heterogeneity

## Abstract

Myocardial ischemia-reperfusion injury (MIRI) remains a major driver of infarct expansion, adverse remodeling, and poor outcomes after reperfusion therapy, yet mechanism-based treatments remain limited. Emerging evidence suggests that MIRI is not simply the additive result of oxidative stress and sterile inflammation. Rather, it reflects a spatiotemporally organized imbalance in immune–cardiac metabolic communication. In this framework, cardiomyocytes, coronary microvascular endothelial cells, fibroblasts, and resident cardiac macrophages act as both injury targets and signal-emitting units. They release succinate, lactate, ATP, lipid mediators, and mitochondrial danger signals. Infiltrating neutrophils, monocytes/macrophages, and lymphocyte subsets decode these cues through reprogramming of glycolysis, oxidative phosphorylation, fatty acid oxidation, and amino acid metabolism. These responses shape inflammatory amplification, resolution, and tissue repair. This review summarizes key connecting mechanisms, including hypoxia sensing, mitochondrial dysfunction, and the axis linking adenosine monophosphate-activated protein kinase (AMPK), sirtuin 1 (SIRT1), and peroxisome proliferator-activated receptor gamma coactivator 1-alpha (PGC-1α). It also discusses immunometabolites such as succinate, lactate, itaconate, and ATP/adenosine as links between tissue injury and immune cell-state transitions. Finally, we highlight temporal windows, spatial niches, and cell-state specificity, and evaluate how single-cell omics, spatial transcriptomics, spatial metabolomics, and metabolic flux analysis may guide time-sensitive, subpopulation-specific, and spatially precise therapies.

## 1 Introduction

Myocardial ischemia-reperfusion injury (MIRI) remains a major determinant of infarct size, adverse cardiac remodeling, and clinical outcome after reperfusion therapy for acute myocardial infarction (1–4). Although timely reperfusion increases myocardial salvage, restoration of blood flow does not necessarily restore tissue homeostasis (5–7). Instead, reperfusion frequently triggers oxidative stress, ion imbalance, microvascular dysfunction, sterile inflammation, and cell death programs that together aggravate secondary myocardial injury (8–12). The conventional view, which largely attributes MIRI to reactive oxygen species (ROS) burst, calcium overload, and inflammatory cascades, explains several core features of the syndrome (7, 8, 13). However, this framework has not translated into effective mechanism-based therapies and does not adequately explain the marked heterogeneity of injury progression and repair across patients, myocardial regions, and disease stages.

Emerging evidence suggests that MIRI is better understood as a disorder of dysregulated immune–cardiac metabolic communication rather than as a simple combination of oxidative and inflammatory injury (14–20). Ischemia and reperfusion induce profound metabolic disturbances in cardiomyocytes, coronary microvascular endothelial cells, fibroblasts, and resident cardiac immune cells (21–26). These cells are not merely passive targets of injury (27). They actively release lactate, succinate, ATP, lipid mediators, mitochondrial danger signals, and other stress-associated molecules, thereby converting local metabolic disruption into signals for immune recruitment, activation, and tissue remodeling (28–33). In parallel, infiltrating neutrophils, monocytes/macrophages, and lymphocytes do not respond passively to these cues. Through dynamic reprogramming of glycolysis, oxidative phosphorylation (OXPHOS), fatty acid oxidation (FAO), and amino acid metabolism, they interpret these tissue-derived signals and thereby shape inflammatory amplification, resolution, and repair (34–36). Accordingly, MIRI should be conceptualized as an imbalance in an immune–cardiac metabolic circuit, rather than as the sum of isolated metabolic alterations in individual cell types.

To improve conceptual clarity, we use the term “immune-cardiac metabolic circuit” as a working framework rather than as a claim that all interactions are already mechanistically resolved. In this review, “signal sources” refer to injured cardiac-resident cells that release metabolites, danger signals, or paracrine mediators, whereas “decoders” refer to immune populations that sense these cues through transporters, receptors, enzymatic pathways, or post-translational metabolic modifications. This terminology is intended to simplify the discussion of cross-cellular immunometabolic communication, not to replace established mechanisms such as oxidative stress, calcium overload, microvascular dysfunction, or regulated cell death.

**Figure 1:**
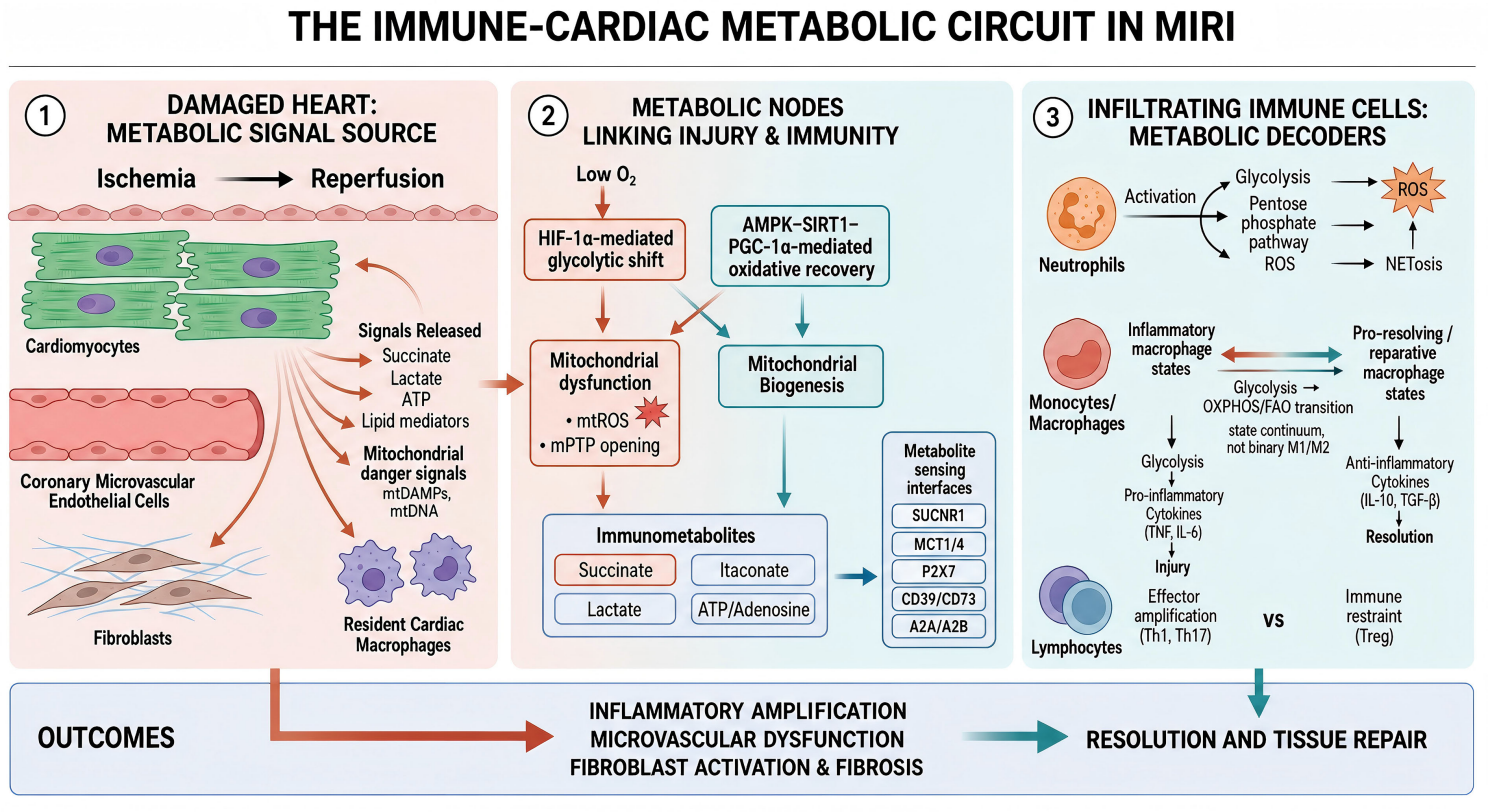
The immune–cardiac metabolic circuit in myocardial ischemia-reperfusion injury. Myocardial ischemia-reperfusion injury (MIRI) can be conceptualized as a reciprocal metabolic circuit between injured cardiac tissue and infiltrating immune cells. After ischemia-reperfusion, cardiomyocytes, coronary microvascular endothelial cells, fibroblasts, and resident cardiac macrophages act as metabolic signal sources that release succinate, lactate, ATP, lipid mediators, and mitochondrial danger signals. These cues are organized through key metabolic nodes, including HIF-1α-dependent glycolytic reallocation, mitochondrial dysfunction, the AMPK–SIRT1–PGC-1α recovery axis, and immunometabolite signaling. Transporters, receptors, enzymatic pathways, and post-translational modifications provide sensing interfaces through which immune cells decode these signals. Neutrophils, monocytes/macrophages, and lymphocytes then adopt distinct metabolic programs that either amplify inflammation and microvascular dysfunction or promote immune restraint, resolution, and reparative remodeling.

This perspective repositions metabolic dysfunction in MIRI from a byproduct of energetic failure to an organized signaling network that links tissue injury, immune cell-state transitions, and repair outcome. Whether reperfusion injury is contained, amplified, or redirected toward coordinated healing depends on three interrelated variables: the nature of metabolic signals released by the injured heart, the metabolic programs engaged by immune cells, and the temporal and spatial context in which these interactions occur. On this basis, this review first considers the injured heart as an active source of metabolic instruction and examines how cardiomyocytes, coronary microvascular endothelial cells, fibroblasts, and resident cardiac macrophages establish the tissue context for subsequent immunometabolic reprogramming. We then discuss how these signals are decoded by infiltrating immune populations and how this process is shaped by time-window dependence, spatial heterogeneity, and cell-state specificity. Viewed in this way, MIRI may be more accurately defined as a spatiotemporally organized disorder of immune–metabolic coupling, a framework that may facilitate the identification of more precise therapeutic targets and translational strategies (**Figure 1**).

To maintain a balanced interpretation, we distinguish throughout the manuscript between established mechanisms, emerging findings, and inferential hypotheses. Established mechanisms include mitochondrial ROS generation, calcium-dependent mPTP opening, neutrophil and monocyte recruitment, and macrophage participation in repair. Emerging findings include single-cell-defined neutrophil and macrophage states, lactylation-dependent injury programs, and spatially restricted cardiomyocyte or stromal states. More inferential areas include lymphocyte metabolic flux in MIRI, receptor-level sensing of some metabolites, and spatial handoff of metabolic signals within the border zone.

## 2 The damaged heart as a source of metabolic signals

In MIRI, the heart should not be regarded merely as a passive target of immune-mediated injury, but as an active source of metabolic signals that shape subsequent immune responses. Ischemia-reperfusion stress induces rapid and profound metabolic remodeling in multiple resident cardiac cell types. Importantly, these changes extend beyond intracellular energetic disturbance. They are translated into intercellular signals through metabolic intermediates, damage-associated molecular patterns (DAMPs), lipid mediators, and inflammatory factors, which in turn drive immune-cell recruitment, activation, and functional polarization. Accordingly, defining how distinct resident cardiac cells generate and transmit these metabolic cues is a necessary first step toward understanding the immune–cardiac metabolic circuit in MIRI.

### 2.1 Cardiomyocytes

Cardiomyocytes serve as both the principal metabolic victims of ischemia-reperfusion and a major source of immune-readable signals in MIRI (37, 38). Under physiological conditions, their contractile activity depends predominantly on mitochondrial oxidative phosphorylation to sustain continuous ATP production. During ischemia, interruption of oxygen and substrate supply shifts cardiomyocyte metabolism from fatty acid and glucose oxidation toward glycolysis, leading to lower energetic efficiency, lactate accumulation, intracellular acidosis, and progressive ionic disequilibrium. Reperfusion does not immediately restore metabolic homeostasis (39). Instead, rapid reoxygenation in the setting of mitochondrial dysfunction promotes electron leakage from the respiratory chain, particularly at complex I, triggers a burst of mitochondrial reactive oxygen species (mtROS), and facilitates calcium overload-dependent opening of the mitochondrial permeability transition pore (mPTP) (32, 33, 40, 41). These events accelerate membrane potential collapse and further ATP depletion. However, this canonical model requires refinement. Recent studies indicate that metabolic rewiring during early reperfusion is not uniformly maladaptive. In cardiomyocytes, limited aerobic glycolysis and regulated lactate handling may transiently support survival, whereas excessive lactate accumulation and lactylation-dependent death pathways may aggravate injury (37, 42). MCT4-dependent lactate export has also been implicated in the regulation of glycolysis–OXPHOS coupling and oxidative stress, suggesting that reperfusion metabolism is better understood as dynamic metabolic reallocation rather than simple energetic failure (43).

The role of cardiomyocytes in MIRI therefore extends beyond bioenergetic collapse. Cardiomyocytes exposed to ischemia-reperfusion convert intracellular metabolic disruption into extracellular signals that can be sensed by immune and vascular cells. Succinate is a representative example. Intracellularly, ischemic succinate accumulation followed by rapid oxidation at reperfusion promotes mtROS generation and mPTP opening, thereby acting as a proximal driver of reperfusion injury (30, 44, 45). Extracellularly, succinate may also function as an inflammatory cue, although its immunological effects appear to be context- and compartment-dependent (45, 46). Lactate should likewise not be regarded merely as a byproduct of glycolytic stress. Emerging evidence suggests that lactate-dependent protein lactylation exerts bidirectional effects in cardiomyocytes: moderate glycolysis-associated lactylation may be adaptive during early reperfusion, whereas excessive lactylation has been linked to NLRP3-dependent pyroptosis and increased susceptibility to ferroptosis through GPX4- or ACSL4-related mechanisms (37, 47–49). ATP release provides a further bridge between metabolic injury and immune regulation. Extracellular ATP acts as an early danger signal, whereas its sequential conversion by CD39 and CD73 generates adenosine, which may attenuate innate immune activation through purinergic signaling (50–52). Collectively, these observations indicate that cardiomyocytes do not simply undergo necrosis and release nonspecific danger signals. Rather, they emit a layered metabolic instruction set whose biological effects depend on timing, localization, and downstream receptor or modification context.

A further advance is the recognition that cardiomyocyte-derived inflammatory signaling cannot be explained solely by passive necrosis (53, 54). Mitochondrial injury can directly engage innate immune machinery within cardiomyocytes. Recent evidence suggests that cardiomyocyte pyroptosis in ischemia-reperfusion injury may be regulated through a PGAM5/MAVS/NLRP3 axis, providing a more specific mechanistic link between mitochondrial stress and inflammatory cell death (55). In parallel, cyclic GMP-AMP synthase (cGAS)–stimulator of interferon genes (STING)-related signaling has increasingly been implicated in the coupling of mitochondrial dysfunction to inflammatory and ferroptotic injury, although its precise cell-specific role in MIRI remains to be fully defined (9, 56). Importantly, cardiomyocytes should not be viewed as a homogeneous source of metabolic stress signals. Recent spatial transcriptomic and single-cell analyses have identified region-specific cardiomyocyte subclusters after myocardial ischemia-reperfusion and have highlighted selective activation of Piezo1-associated calcium dysregulation programs in distinct cardiomyocyte states (57). These findings suggest that the metabolic signals emitted by cardiomyocytes are both spatially stratified and state dependent (57). Within the immune–cardiac metabolic circuit, cardiomyocytes therefore function not only as metabolically injured cells, but also as context-specific signal-emitting units that shape the magnitude, location, and trajectory of downstream inflammation and repair.

### 2.2 Coronary microvascular endothelial cells

Coronary microvascular endothelial cells function as early signal-transducing units that convert ischemia-reperfusion stress into immune recruitment and microvascular dysfunction in MIRI (58, 59). Unlike cardiomyocytes, endothelial cells rely predominantly on glycolysis under basal conditions (60). However, this metabolic profile does not confer resistance to reperfusion injury. During reperfusion, endothelial cells develop mitochondrial dysfunction, impaired oxidative phosphorylation, and redox imbalance (61–63). Glycolysis is further enhanced to sustain basal ATP demand, but this compensatory adaptation is frequently accompanied by endothelial nitric oxide synthase uncoupling, reduced nitric oxide bioavailability, and excessive reactive oxygen species generation (60). Consequently, vasodilatory capacity declines, barrier integrity is disrupted, and microvascular perfusion deteriorates (64). Recent evidence further suggests that endothelial mitochondrial quality control, including FUNDC1-dependent mitophagy, is a key determinant of whether reperfusion stress remains contained or progresses to structural and inflammatory microvascular injury (65).

The pathological significance of endothelial metabolic remodeling lies in its capacity to translate local metabolic stress into pro-adhesive, pro-inflammatory, and no-reflow-promoting signals (5). Metabolically stressed endothelial cells acquire a phenotype characterized by reduced nitric oxide signaling, increased endothelin-1 activity, destabilization of VE-cadherin-dependent junctions, and activation of adhesion-related programs that facilitate neutrophil and monocyte adhesion, rolling, and transmigration (66–69). More recent studies have clarified the molecular basis of this transition. Endothelial S1PR2 has been shown to aggravate ischemia-reperfusion injury through a RHO/ROCK1/DRP1 axis that promotes mitochondrial fission, oxidative stress, NLRP3 activation, and pyroptosis (70). In parallel, the endothelial HMGB1-AIM2 axis has been implicated in barrier disruption and inflammatory amplification, and single-cell analyses have suggested dynamic changes in a FABP4-high endothelial subpopulation after injury (71). Together, these findings indicate that endothelial dysfunction in MIRI cannot be explained by oxidative stress alone. Rather, mitochondrial stress, inflammasome signaling, and inflammatory endothelial cell death form a mechanistic bridge between metabolic disturbance and immune cell recruitment (72).

Importantly, coronary microvascular endothelial cells should not be viewed solely as passive amplifiers of tissue injury. Under certain conditions, they may also generate signals that restrain injury propagation. Endothelial cell-derived exosomal sphingosylphosphorylcholine has been reported to attenuate myocardial ischemia-reperfusion injury through NR4A2-mediated mitophagy (73). By contrast, endothelial ferroptosis has emerged as an additional mechanism of microvascular injury, while activation of the PEDF/34-mer–Nrf2/HO-1 pathway can partially preserve endothelial integrity and perfusion (74). Taken together, these observations support a more nuanced view of coronary microvascular endothelial cells as context-dependent signaling units. Their central role in MIRI is not merely to reflect vascular injury, but to determine leukocyte access, vascular tone, barrier stability, and the extent to which local metabolic stress is propagated across the injured myocardium.

### 2.3 Fibroblasts

Cardiac fibroblasts act as metabolically regulated organizers of the post-ischemic reparative niche in MIRI (75). Their activation is not driven by cytokine signaling alone, but also by coordinated metabolic rewiring that supports myofibroblast transition and extracellular matrix (ECM) production. Following reperfusion injury, fibroblasts typically increase glycolytic flux to sustain proliferation, migration, and matrix synthesis, a program supported by PFKFB3-dependent glycolytic activation (76). In parallel, glutamine metabolism can replenish tricarboxylic acid cycle intermediates and support anabolic pathways, whereas proline and hydroxyproline metabolism provides substrates for collagen deposition (77). This adaptive program may initially stabilize the injured myocardium; however, if it persists, it promotes excessive ECM accumulation, tissue stiffening, and adverse remodeling. Recent studies further indicate that this process is mechanistically organized rather than nonspecific. PFKFB3 has emerged as an important driver of fibroblast glycolytic activation and myofibroblast differentiation, while restoration of autophagic flux, including VMP1-dependent inhibition of mTOR signaling, restrains fibroblast activation and attenuates post-ischemic fibrosis (78–80). These observations suggest that fibroblast fate is governed by the balance between anabolic drive and metabolic quality control.

The importance of fibroblast metabolic remodeling extends beyond collagen synthesis. Activated fibroblasts reshape the inflammatory milieu by altering lactate availability, ECM composition, tissue stiffness, and the local paracrine environment. In turn, these changes influence immune cell retention, macrophage polarization, and the persistence of reparative versus pro-fibrotic programs. Recent single-cell studies in MIRI support this concept by showing that the transition from acute inflammation to fibrosis is associated with a distinct S100a9^hi macrophage state that activates TGF-β/p-Smad3 signaling and promotes fibroblast-to-myofibroblast transition, as well as macrophage-to-myofibroblast transition (81). Thus, fibroblast activation should not be viewed simply as a terminal downstream consequence of inflammation. Rather, fibroblasts and inflammatory myeloid cells form a reciprocal circuit that establishes and maintains a pro-fibrotic niche. This interpretation is further supported by spatial transcriptomic studies showing marked regional heterogeneity between infarct core and surrounding myocardium after ischemia-reperfusion, indicating that fibroblast responses are spatially stratified rather than uniform (57, 82).

Accordingly, fibroblasts should be incorporated into the immune–cardiac metabolic circuit as context-dependent signal-emitting and niche-shaping cells, rather than passive matrix producers. By coupling glycolytic and anabolic rewiring to ECM remodeling, autophagy status, and immune-active paracrine output, fibroblasts help determine whether the post-ischemic myocardium progresses toward inflammation resolution and scar stabilization or toward persistent inflammation and pathological fibrosis (83, 84). In this sense, fibroblasts represent a critical decision node linking metabolic adaptation, immune regulation, and chronic structural remodeling.

### 2.4 Resident cardiac macrophages

Resident cardiac macrophages function as local metabolic and immunological organizers that help preserve tissue homeostasis and coordinate repair after myocardial ischemia-reperfusion injury. Under physiological conditions, they support tissue surveillance, apoptotic cell clearance, electrical stability, and local immune tolerance. These functions are coupled to metabolic programs that favor efferocytosis and tissue adaptation rather than acute inflammatory activation. Following ischemia-reperfusion, resident macrophages are among the earliest immune cells exposed to hypoxia, extracellular ATP, lipid mediators, and signals derived from injured cardiomyocytes. Their importance therefore lies not only in their intrinsic phenotype, but also in their ability to shape the subsequent immune environment. Recent studies indicate that resident and recruited macrophages make distinct contributions to post-ischemic healing (85). Notably, depletion of resident macrophages does not necessarily alter infarct size, but it disrupts immune cell crosstalk, promotes pro-inflammatory neutrophil polarization, and worsens cardiac remodeling (85). These findings suggest that resident macrophages should be viewed less as a static anti-inflammatory subset and more as upstream coordinators of inflammatory resolution.

This interpretation is also consistent with foundational macrophage studies outside strict MIRI models. Fate-mapping and single-cell analyses showed that self-renewing TIMD4+LYVE1+ resident cardiac macrophages limit adverse remodeling after myocardial infarction, whereas time-course transcriptomics demonstrated that macrophage polarization changes along a continuum rather than a binary M1/M2 switch (86, 87). More recent single-cell multiomic and functional-diversity studies further support the view that macrophage subsets occupy distinct reparative or inflammatory niches (88). Because some of these studies used permanent infarction rather than ischemia-reperfusion, their conclusions should be extrapolated to MIRI with appropriate caution.

In MIRI, resident macrophage reprogramming should not be reduced to a simple oxidative-to-glycolytic shift. Its key significance lies in the ability of resident macrophages to shape downstream myeloid trajectories and set the local threshold for inflammatory resolution. Detailed HIF-1α-dependent mechanisms of monocyte fate specification are discussed in Section 4.1. Here, the central point is that resident macrophages influence repair both through their own efferocytic programs and through non-cell-autonomous regulation of recruited macrophage differentiation.

A further advance is the recognition that the reparative activity of resident macrophages is tightly linked to metabolically sustained efferocytosis. Recent studies have identified Sectm1a as a macrophage-enriched regulator of efferocytosis that enhances GITR/LXRα signaling and promotes expression of engulfment- and lysosome-associated genes during ischemia-reperfusion injury (89). More recently, Maresin 1 was shown to protect against myocardial ischemia-reperfusion injury by selectively enhancing efferocytosis in resident macrophages (90). Mechanistically, this effect was associated with increased fatty acid β-oxidation and activation of a PPARγ–CD204 axis, whereas genetic depletion of resident macrophages abolished Maresin 1-mediated cardioprotection (90). Together, these findings indicate that resident cardiac macrophages are not merely repair-associated in a descriptive sense. Rather, they rely on defined metabolic programs, particularly FAO-coupled efferocytosis, to restrain inflammation and support tissue recovery.

Accordingly, the significance of resident cardiac macrophages in the immune–cardiac metabolic circuit lies in their dual role as metabolic templates of homeostasis and gatekeepers of reparative reprogramming. When oxidative and efferocytic programs are suppressed by sustained pro-inflammatory pressure, the injured heart loses an endogenous mechanism that normally limits neutrophil polarization, guides monocyte differentiation, and facilitates timely resolution. By contrast, preservation or restoration of FAO-linked efferocytosis and hypoxia-adaptive signaling in resident macrophages may provide a more selective strategy to re-establish the balance between inflammation and repair (91, 92). Resident cardiac macrophages should therefore not be regarded as a minor macrophage subset, but as a decisive regulatory node linking local metabolic stress to downstream immune fate and remodeling outcome.

## 3 Infiltrating immune cells as metabolic decoders

In MIRI, the damaged heart does not automatically progress toward a specific inflammatory or reparative outcome. What truly determines the course of the disease is how infiltrating immune cells perceive and decode metabolic signals from ischemic-reperfused myocardium (93), including a local stress environment characterized by hypoxia, lactate accumulation, succinate release, ATP leakage, ROS bursts, and necrotic cell debris (28–33). Different interpretations of these signals drive distinct immune cells into radically different metabolic pathways: some cells rapidly shift toward hyperglycolysis and oxidative burst, thereby amplifying acute injury; others gradually transition to a state dominated by OXPHOS and FAO in the later stages, thereby supporting the resolution of inflammation and tissue repair (21–26). Thus, infiltrating immune cells in MIRI are not passive executors of inflammation, but rather key functional decoders of cardiac metabolic stress.

### 3.1 Neutrophils

Neutrophils are early metabolic decoders of myocardial stress in MIRI rather than merely the first leukocytes to infiltrate the injured heart (94). Following reperfusion, local chemokines, complement fragments, hypoxia, extracellular ATP, and oxidative stress rapidly shift neutrophils toward a highly glycolytic state. At the same time, the pentose phosphate pathway is enhanced to provide NADPH for NOX2-dependent oxidant generation (95). This coordinated glycolytic–PPP program supports migration, degranulation, and oxidative burst, thereby converting tissue-derived metabolic stress into direct inflammatory injury (96). Although mitochondria are not the principal source of ATP in neutrophils, they remain functionally important in this setting. Calcium imbalance and mitochondrial reactive oxygen species amplify oxidant signaling and lower the threshold for neutrophil extracellular trap (NET) formation (97). Thus, the pathogenic role of neutrophils in early MIRI is best understood as a coupled glycolytic, redox, and NETotic response to local metabolic danger signals.

Recent work, however, indicates that neutrophil responses in MIRI are not metabolically uniform. Single-cell studies have identified a cardioprotective Ym-1^hi neutrophil subset that emerges early after MIRI and appears to favor macrophage polarization toward a reparative state (98). In parallel, a distinct neutrophil population enriched for type I interferon signaling has been shown to depend on neutrophil-intrinsic STING activity; conversely, neutrophil-specific deletion of STING worsens cardiac function and disrupts downstream myeloid responses (98). These findings revise the conventional view that neutrophils are uniformly deleterious in MIRI. Instead, neutrophils should be regarded as a heterogeneous population composed of temporally and functionally distinct states, some of which amplify tissue injury, whereas others contribute to inflammatory resolution. This distinction has direct conceptual implications, because non-selective neutrophil suppression may eliminate both damaging and protective subsets.

A further advance has been the identification of lactylation-dependent neutrophil programming in MIRI (34). Lactyl-proteomic analysis showed that S100A9 undergoes lysine-26 lactylation in neutrophils in a DLAT-dependent manner (34). Functionally, lactylated S100A9 promoted transcription of migration-associated genes, enhanced cardiac neutrophil recruitment, and was released during NET formation, where it contributed to cardiomyocyte mitochondrial dysfunction and cell death (34). In patients with acute myocardial infarction, elevated circulating S100A9 lactylation was associated with poorer outcome, supporting the translational relevance of this pathway. These findings extend the conventional glycolysis–PPP–ROS framework by showing that lactate-dependent protein modification is not merely a metabolic byproduct, but a mechanistic link between neutrophil metabolic rewiring, tissue trafficking, and downstream injury (99). Accordingly, the role of neutrophils in the immune–cardiac metabolic circuit lies not only in rapid pro-inflammatory activation, but also in the state-dependent decoding of the same metabolic environment into distinct outcomes for inflammation, microvascular dysfunction, and repair.

### 3.2 Monocytes/macrophages

Monocytes and macrophages are the most metabolically plastic immune cells in MIRI and are central to determining whether post-ischemic inflammation is amplified, redirected, or resolved (100, 101). Rather than fitting a rigid M1/M2 framework, macrophage responses in MIRI are better understood as dynamic transitions along metabolic and transcriptional trajectories. This view is supported by time-resolved mapping of macrophage polarization after myocardial infarction and by single-cell studies showing that resident and recruited macrophages occupy overlapping but non-redundant functional states (86–88, 102). During early reperfusion, recruited monocytes and monocyte-derived macrophages adopt a highly glycolytic and inflammatory state in response to hypoxia, lactate accumulation, necrotic debris, and inflammatory mediators. This program is characterized by increased glucose utilization, HIF-1α stabilization, pentose phosphate pathway activation, enhanced inducible nitric oxide synthase-associated arginine metabolism, and sustained production of IL-1β, TNF-α, and reactive oxygen species. Nevertheless, this description should be treated as a functional approximation rather than a fixed phenotype, because macrophage states vary by ontogeny, location, and time after injury. Importantly, recent single-cell studies indicate that this early inflammatory compartment is not uniform (16). A distinct S100a9^hi monocyte-derived macrophage subset has been identified in myocardial ischemia-reperfusion injury, appearing within hours after reperfusion and amplifying acute inflammatory signaling through a MyD88/NF-κB/NLRP3 axis (53). These findings suggest that macrophage-driven injury in early MIRI is better attributed to specific inflammatory states than to a generic “M1-like” population.

As reperfusion progresses, macrophage metabolism may shift from glycolysis-dominant inflammatory activation toward reparative programs supported by oxidative phosphorylation and fatty acid oxidation. This transition is typically associated with enhanced efferocytosis, restoration of mitochondrial function, and activation of PPARγ/PGC-1-related networks. At this stage, macrophages are more likely to support apoptotic cell clearance, limit inflammatory persistence, promote angiogenesis, and stabilize extracellular matrix remodeling. However, this reparative phase is also heterogeneous. Single-cell analysis has identified a distinct SPP1^+ lipid-associated macrophage (LAM) subset in myocardial ischemia-reperfusion injury, enriched for lipid metabolic programs and MAPK-related signaling, indicating that reparative macrophage states are metabolically diverse rather than uniform (103). In parallel, recent mechanistic evidence has shown that PGC-1α restrains pro-inflammatory macrophage polarization and mitigates myocardial ischemia-reperfusion injury through dual regulation of SUCLG1/succinate metabolism and TRAF5 expression, thereby directly linking oxidative metabolic recovery to inflammatory restraint (104). Likewise, itaconate-related pathways should not be viewed simply as generic anti-inflammatory signatures. 4-octyl itaconate has been shown to attenuate myocardial ischemia-reperfusion injury and promote angiogenesis, supporting the concept that macrophage-associated metabolic buffering also contributes to reconstruction of the reparative microenvironment (105).

The significance of monocytes/macrophages in MIRI therefore extends beyond a simple transition from inflammatory to reparative phenotypes. These cells act as major redistributors of metabolic information across the injured heart. They sense lactate, succinate, ATP/adenosine, and necrotic debris, and they in turn reshape fibroblast activation, vascular remodeling, and subsequent lymphocyte responses through cytokines, metabolites, and lipid mediators (106). Recent work has made this redistributive role more explicit. The S100a9^hi macrophage state not only amplifies acute inflammation, but also promotes progression toward fibrosis through TGF-β/p-Smad3 signaling and macrophage-to-myofibroblast transition (53). Similarly, GM-CSF/CCL2/CCR2 signaling has been shown to accelerate the progression from acute injury to cardiac fibrosis by engaging NLRP3/caspase-1/IL-1β signaling, promoting macrophage state switching, and increasing macrophage-derived TGF-β release to fibroblasts (107). Accordingly, monocytes and macrophages in MIRI should be viewed not only as metabolic decoders of tissue stress, but also as key redistributors that determine whether the immune–cardiac metabolic circuit remains locked in injury amplification or proceeds toward controlled resolution and repair.

### 3.3 Lymphocytes

Lymphocytes in MIRI are best conceptualized as delayed metabolic decoders that partition immune responses between effector amplification and regulatory restraint (108). Compared with myeloid cells, direct evidence for lymphocyte metabolic rewiring in myocardial ischemia-reperfusion remains limited. Nevertheless, the available data support a framework in which distinct lymphocyte subsets engage different metabolic programs that influence whether post-ischemic inflammation persists or resolves (109). In general, activated effector T cells, including Th1, Th17, and CD8^+ T cells, are expected to depend preferentially on glycolysis and glutamine utilization to sustain proliferation and the production of IFN-γ, IL-17, and cytotoxic mediators. In the setting of MIRI, such programs are likely to reinforce inflammatory crosstalk with macrophages and activated fibroblasts, thereby prolonging tissue injury and adverse remodeling (110). However, this interpretation remains inferential to a considerable extent, because direct metabolic flux-based evidence for effector T-cell programming in MIRI is still sparse.

By contrast, regulatory lymphocyte subsets are more closely associated with oxidative metabolic programs and the stabilization of inflammatory resolution. Regulatory T cells (Tregs) are generally supported by oxidative phosphorylation and fatty acid oxidation, and this principle is likely relevant to MIRI, particularly during the reparative phase and within the border zone, where sustained glycolytic effector responses may be less favorable (2). Accordingly, Tregs in MIRI should be viewed not simply as anti-inflammatory cells, but as metabolic regulators of repair-supportive immune restraint. Yet here again, direct metabolic measurements remain limited, and current interpretation rests largely on functional association rather than definitive flux analysis. A similar degree of caution applies to B cells. Although activated B cells are generally expected to increase glycolysis and glutamine metabolism during antibody-producing differentiation, direct metabolic evidence in MIRI is lacking. The clearest recent evidence instead concerns regulatory B-cell function. T-cell immunoglobulin and mucin domain 1 (TIM1)^+ regulatory B cells (Bregs) were shown to attenuate myocardial ischemia-reperfusion injury by increasing IL-10 production, suppressing inflammatory T-cell responses, and promoting Treg activation (111). These findings suggest that part of the lymphocyte compartment participates in a metabolically permissive restraining circuit, although the precise metabolic architecture of Bregs in MIRI remains unresolved.

Other lymphocyte populations, including natural killer T (NKT) cells and γδ T cells, may also contribute to immune regulation in MIRI, but their metabolic basis remains poorly defined (112). At present, they should therefore be regarded as emerging rather than established components of the MIRI immunometabolic landscape. Taken together, lymphocytes in MIRI are most usefully understood not by absolute changes in subset abundance, but by which metabolic logic dominates at a given stage after reperfusion. Effector T-cell programs are more likely to sustain inflammatory crosstalk, whereas Tregs and TIM1^+ Bregs appear to support immune braking and repair (111). In this sense, lymphocytes function as delayed but consequential metabolic decoders whose impact depends less on cell number alone than on the balance between glycolytic effector persistence and oxidative immune restraint.

## 4 Key metabolic nodes linking signal sources and decoders

The ability of a damaged heart to drive subsequent immune responses stems not merely from the release of additional inflammatory factors, but from the fact that ischemia and reperfusion jointly reshape a set of metabolic nodes capable of crossing cellular boundaries. These nodes determine, on the one hand, how cardiomyocytes, endothelial cells, and fibroblasts transmit danger signals, and on the other hand, which metabolic pathways neutrophils, monocytes/macrophages, and lymphocytes will follow. In other words, what truly merits attention in MIRI is not which pathways are activated, but which metabolic nodes serve as the interface between the cardiac signal source and the immune decoders.

### 4.1 Hypoxia sensing and glycolytic reallocation

HIF-1α in MIRI is best regarded as a state-defining regulator that allocates metabolic and functional responses across distinct cell types, rather than as a passive marker of hypoxic stress (113). During ischemia and early reperfusion, tissue hypoxia, reactive oxygen species, and accumulated metabolites converge to stabilize HIF-1α and induce a glycolytic transcriptional program, including upregulation of GLUT1, HK2, PFKFB3, LDHA, and PDK1. In inflammatory neutrophils and monocyte-derived macrophages, this program promotes glucose uptake, pentose phosphate pathway engagement, oxidant production, and pro-inflammatory cytokine release, thereby linking local metabolic stress to acute tissue injury. However, HIF-1α should not be interpreted as a uniformly pro-inflammatory switch. In cardiomyocytes, HIF-1α-dependent maintenance of aerobic glycolysis during early reperfusion can be adaptive. HSPA12A has been shown to stabilize HIF-1α, preserve aerobic glycolytic flux, sustain histone H3 lactylation, and thereby improve cardiomyocyte survival after myocardial ischemia-reperfusion. These findings indicate that the biological role of HIF-1α depends on cellular context and stage of injury, rather than on hypoxia alone (114).

Recent evidence further suggests that the importance of HIF-1α in MIRI extends beyond direct induction of glycolytic genes. Hypoxia sensing through HIF-1α in resident cardiac macrophages was shown to regulate monocyte fate specification after ischemic injury (115). Deletion of Hif1a in resident macrophages did not simply increase inflammatory activation. Instead, it disrupted monocyte differentiation trajectories and led to accumulation of an Arg1^+ inflammatory intermediate state associated with worsened remodeling (115). This finding substantially revises the conventional view that HIF-1α acts only by reinforcing inflammatory metabolism within individual immune cells. Rather, it indicates that HIF-1α can also shape downstream myeloid-state transitions in a non-cell-autonomous manner. Accordingly, HIF-1α should be interpreted as a context-dependent allocation node: depending on cell type and injury phase, it can translate hypoxic stress into inflammatory amplification, adaptive metabolic buffering, or reparative progression.

### 4.2 Mitochondrial dysfunction and ROS

Mitochondria amplify reperfusion injury by converting metabolic disequilibrium into oxidant stress, inflammatory signaling, and regulated cell death. During ischemia, succinate accumulates as a consequence of interrupted oxidative metabolism. Upon reperfusion, rapid oxidation of this pool drives a burst of mitochondrial reactive oxygen species (mtROS), promotes mitochondrial permeability transition pore (mPTP) opening, and accelerates membrane potential collapse. Importantly, recent evidence suggests that the severity of injury depends not simply on the presence of succinate, but on its dynamic intracellular and extracellular distribution (116, 117). Thus, mitochondrial dysfunction in MIRI should be understood not merely as energetic failure, but as an active signaling process through which metabolic mismatch is translated into structural and inflammatory injury. This process is also spatially heterogeneous. Recent spatial transcriptomic analyses indicate that infarct core regions are preferentially enriched for ferroptosis- and mitophagy-related programs, supporting the view that mitochondrial stress is not uniformly distributed across the reperfused myocardium (118).

Persistent mitochondrial stress subsequently lowers the threshold for inflammasome activation and inflammatory cell death. In cardiomyocytes, recent studies have defined a more specific mechanistic link between mitochondrial dysfunction and pyroptosis. MARCH2 has been shown to protect against myocardial ischemia-reperfusion injury by suppressing cardiomyocyte pyroptosis through negative regulation of the PGAM5/MAVS/NLRP3 axis, and single-cell analysis further suggests that loss of MARCH2 promotes NLRP3 activation in cardiomyocytes (55). In parallel, NEK7/NLRP3 signaling has emerged as an additional interface linking oxidative stress to inflammasome assembly and cytokine release (119). These findings indicate that mtROS and mitochondrial damage do not merely correlate with inflammasome activation, but can be translated into defined pyroptotic execution pathways.

Recent work further shows that mitochondrial dysfunction in MIRI extends beyond pyroptosis to ferroptotic injury. cGAS–STING signaling was shown to aggravate myocardial ischemia-reperfusion injury by promoting autophagic degradation of GPX4, thereby coupling mitochondrial stress to ferroptosis in cardiomyocytes (120). This observation expands the conventional mtROS–NLRP3 framework by showing that mitochondrial danger signaling can be directed toward distinct downstream death programs depending on molecular context and cell type. Taken together, these findings support a revised view of mitochondria in MIRI: they are not simply the earliest organelles to fail, but a central signaling hub that redistributes metabolic stress into pyroptosis, ferroptosis, inflammatory cytokine production, and spatially organized tissue injury.

### 4.3 The AMPK–SIRT1–PGC-1α axis

The adenosine monophosphate-activated protein kinase (AMPK)–sirtuin 1 (SIRT1)–peroxisome proliferator-activated receptor gamma coactivator 1-alpha (PGC-1α) axis in MIRI is best viewed as a metabolic reconstruction network that restores oxidative homeostasis while limiting inflammatory persistence. In contrast to HIF-1α, which preferentially supports stress-adaptive glycolysis, this axis promotes mitochondrial recovery, rebalances substrate utilization, and re-establishes post-reperfusion metabolic stability. AMPK is a key upstream sensor in this process. Recent studies have shown that AMPK activation attenuates myocardial ischemia-reperfusion injury by suppressing DRP1-dependent mitochondrial fission, reducing reactive oxygen species generation, and lowering inflammatory cytokine expression (121). In the cardiac microvasculature, AMPKα1/ULK1/FUNDC1-dependent mitophagy has also been shown to preserve endothelial integrity, reduce neutrophil infiltration, and limit microvascular hyperpermeability after reperfusion (65).These findings indicate that AMPK-mediated protection is not merely a matter of energetic support, but a mechanism that links mitochondrial quality control to inflammatory limitation. SIRT1 and PGC-1α further reinforce this program by supporting deacetylation-dependent stress adaptation, mitochondrial biogenesis, and re-engagement of oxidative phosphorylation and fatty acid oxidation. Accordingly, the AMPK-SIRT1-PGC-1α axis should be understood not as a generic survival pathway, but as a coordinated system of metabolic repair.

Recent work has further extended the significance of this axis from myocardial protection to immune-state regulation. PGC-1α was shown to suppress pro-inflammatory macrophage polarization and mitigate myocardial ischemia-reperfusion injury through dual regulation of SUCLG1/succinate metabolism and TRAF5 expression (104). This finding is important because it demonstrates that oxidative metabolic recovery is not merely a downstream consequence of inflammation resolution, but can actively drive the transition away from inflammatory persistence. In macrophages, therefore, the AMPK–SIRT1–PGC-1α network contributes not only to mitochondrial restoration, but also to restraint of TLR4/NF-κB-associated inflammatory programming. This substantially refines the mechanistic significance of the axis in MIRI: it does not simply improve energy metabolism, but helps determine whether myeloid cells remain trapped in a glycolysis-dominant inflammatory state or progress toward oxidative, pro-resolving programs. Conversely, delayed or unstable reactivation of this network may prolong mitochondrial fragmentation, reactive oxygen species signaling, and inflammatory myeloid persistence, thereby contributing to failed repair. Taken together, these findings support a revised view in which the AMPK-SIRT1-PGC-1α axis serves as a central interface linking mitochondrial recovery, immunometabolic reprogramming, and tissue-level resolution after reperfusion (122).

### 4.4 Metabolic intermediates as circuit nodes

Metabolic intermediates in MIRI function not merely as byproducts of cellular stress, but as circuit nodes that externalize intracellular metabolic states into intercellular signals. Succinate provides the clearest example of this principle. During ischemia, succinate accumulates intracellularly and, upon reperfusion, its rapid oxidation drives mitochondrial reactive oxygen species generation and promotes mPTP opening. At the same time, a fraction of accumulated succinate can be exported from the injured myocardium, allowing intracellular mitochondrial injury and extracellular immune signaling to coexist. The transporter- and receptor-level interfaces of this process are discussed in Section 4.5. Importantly, recent evidence suggests that the impact of succinate depends less on its absolute abundance than on the balance between intracellular retention and extracellular release. Inhibition of succinate efflux aggravates myocardial reperfusion injury by increasing intracellular succinate availability for mitochondrial oxidation and oxidant production. Thus, succinate should be understood not simply as a pro-inflammatory metabolite, but as a dynamically partitioned node that links myocardial metabolic crisis to both intracellular oxidative injury and extracellular immune signaling.

The biological role of lactate is more strongly conditioned by dose, source, and context. Although lactate has long been regarded as a marker of glycolytic stress and tissue acidification, recent evidence indicates that it also functions as a signaling metabolite with both adaptive and maladaptive effects. Lactate was shown to contribute to remote ischemic preconditioning-mediated cardioprotection by promoting autophagy through the AMPK–mTOR–TFEB–Cx43 axis (123). By contrast, more recent work suggests that excessive glycolysis-derived lactate can aggravate MIRI by promoting lactylation-dependent ferroptotic programs, including GPX4 lactylation and, more recently, ACSL4 lactylation (48, 49). These observations substantially revise the conventional view of lactate as a passive glycolytic byproduct. Instead, lactate should be regarded as a context-dependent circuit node whose effects are increasingly mediated through lactylation-dependent remodeling of stress adaptation and cell death pathways.

Itaconate represents a distinct type of metabolic node and functions primarily as an endogenous buffering signal. As a product of the IRG1/ACOD1 pathway, itaconate is linked to succinate dehydrogenase inhibition, attenuation of oxidative stress, and restriction of pro-inflammatory transcriptional programs. In MIRI, however, its significance extends beyond inflammatory suppression alone. 4-octyl itaconate (4-OI) was shown to attenuate myocardial ischemia-reperfusion injury while promoting angiogenesis, indicating that itaconate can bias the tissue microenvironment toward reparative remodeling rather than merely dampening inflammation. Accordingly, within the immune–cardiac metabolic circuit, itaconate is best viewed as a resolution-promoting node that helps shift the system from injury amplification toward controlled repair.

The ATP–adenosine axis provides a particularly clear example of how a single metabolic pathway can encode opposite biological meanings across different phases of injury. During ischemia and reperfusion, extracellular ATP/ADP released from damaged tissue and activated immune cells acts as an early danger signal and amplifies inflammatory sensing, including P2X7-associated signaling. As reperfusion progresses, extracellular adenine nucleotides can be enzymatically converted into adenosine, shifting purinergic signaling from danger amplification toward immune restraint. The relevant enzymatic and receptor interfaces are discussed in Section 4.5. This transition also has direct translational relevance. Hydrogel-based local delivery of CD39/CD73 reduced neutrophil activation, NETosis, innate immune infiltration, and post-MIRI functional decline (51). Thus, the ATP–adenosine axis does not simply illustrate metabolite conversion; rather, it exemplifies how one purinergic circuit can mediate both early danger amplification and later inflammatory restraint, depending on enzymatic context and timing (124).

Taken together, succinate, lactate, itaconate, and ATP–adenosine should not be treated as isolated “classical metabolites, ” but as interface molecules that convert intracellular metabolic states into microenvironmental signals. Their significance in MIRI lies precisely in this function: they propagate danger, buffer injury, or promote resolution according to their cellular origin, biochemical conversion, and spatiotemporal context. In this sense, metabolic intermediates are not peripheral features of the immune–cardiac metabolic circuit, but central nodes through which metabolic crisis is transmitted, interpreted, and potentially redirected.

### 4.5 Metabolite sensing and actionable receptor interfaces

A critical translational question is not only which metabolites accumulate, but how recipient cells sense them. Succinate can leave injured myocardium through MCT1-dependent efflux and may signal through SUCNR1/GPR91; however, its intracellular retention also fuels mitochondrial oxidation and ROS generation, meaning that indiscriminate inhibition of succinate handling may aggravate rather than attenuate injury (45, 116, 117). Lactate can influence cells through monocarboxylate transporters, local acidification, hydroxycarboxylic acid receptor 1/GPR81 signaling, and protein lactylation, but in MIRI the strongest direct evidence currently concerns MCT4-dependent export and lactylation-mediated changes in cardiomyocyte or neutrophil function (34, 37, 43, 47–49). The ATP-adenosine axis is more pharmacologically mature because extracellular ATP can activate P2X7-associated inflammatory sensing, whereas CD39/CD73-dependent adenosine generation can engage A2A/A2B receptor-mediated restraint (50–52). Itaconate and 4-OI appear to act mainly through intracellular redox and electrophilic mechanisms, including SDH- and Nrf2-related programs, rather than through a single established extracellular receptor (105, 124). These distinctions suggest that future therapy should prioritize the transporter, receptor, enzyme, or post-translational modification that is active in a defined cell state, lesion site, and time window, rather than globally increasing or depleting a metabolite.

**Table 1** summarizes the major metabolic signal sources, immune decoders, sensing interfaces, functional consequences, and key references in MIRI.

**Table 1:** Major metabolic signal sources, immune decoders, and sensing interfaces in MIRI.

Signal source or niche	Major metabolic cue	Main sensing/action interface	Principal decoder or responding cell	Main consequence in MIRI	Key references
Stressed cardiomyocytes	Succinate	Mitochondrial oxidation; succinate efflux; possible SUCNR1/GPR91 sensing	Cardiomyocytes, endothelial cells, myeloid cells	Drives mtROS generation and mPTP opening; may also act as an extracellular inflammatory cue	(30, 44, 45, 116, 117)
Stressed cardiomyocytes and glycolytic inflammatory niches	Lactate and lactylation	MCT4-dependent export; protein or histone lactylation; possible lactate receptor signaling	Cardiomyocytes, neutrophils, macrophages	Limited lactate-associated adaptation may support survival, whereas excessive lactylation may promote pyroptosis, ferroptosis, or neutrophil trafficking	(34, 37, 43, 47–49, 123)
Injured tissue and activated immune cells	ATP/adenosine	P2X7-associated ATP sensing; CD39/CD73-mediated adenosine generation; A2A/A2B signaling	Neutrophils, macrophages, endothelial cells, cardiomyocytes	ATP amplifies early danger signaling; adenosine promotes immune restraint and cardioprotection	(50–52, 124)
Damaged mitochondria in cardiomyocytes and vascular cells	mtROS, mtDNA, mitochondrial DAMPs	mPTP opening; PGAM5/MAVS/NLRP3; NEK7/NLRP3; cGAS-STING-GPX4 axis	Cardiomyocytes, macrophages, endothelial cells	Converts metabolic mismatch into pyroptosis, ferroptosis, inflammatory cytokine release, and tissue injury	(9, 32, 33, 40, 41, 53–56, 119, 120)
Coronary microvascular endothelial cells	Redox imbalance, NO deficiency, barrier-disruptive signals	eNOS uncoupling; VE-cadherin disruption; S1PR2-RHO/ROCK1/DRP1; HMGB1-AIM2; FUNDC1 mitophagy	Neutrophils, monocytes, endothelial inflammatory states	Controls leukocyte entry, no-reflow, barrier dysfunction, and microvascular hyperpermeability	(58, 59, 65, 70–74)
Activated fibroblasts/stromal niche	Lactate availability, ECM stiffness, fibrotic paracrine signals	PFKFB3 glycolysis; VMP1-mTOR/autophagy; TGF-β/p-Smad3; ECM mechanotransduction	Macrophages, fibroblast/myofibroblast states, T cells	Shapes immune retention, fibrosis, scar stabilization, or adverse remodeling	(57, 75–81, 83, 84)
Resident cardiac macrophages	Efferocytosis-linked lipid handling and pro-resolving programs	Sectm1a-GITR/LXRα; HIF-1α-dependent monocyte fate control; Maresin 1-FAO-PPARγ-CD204	Recruited monocytes/macrophages, neutrophils, resident macrophages	Coordinates efferocytosis, inflammatory resolution, monocyte differentiation, and reparative reprogramming	(85–93, 115)
Infiltrating myeloid cells	Glycolysis, PPP activation, S100A9, itaconate-related buffering	NOX2-dependent oxidant generation; NETosis; S100A9 lactylation; PGC-1α-SUCLG1/TRAF5; 4-OI/IRG1-related pathways	Neutrophil subsets; S100a9^hi and SPP1^+ macrophage states	Determines whether infiltrating myeloid cells amplify acute injury or shift toward resolution and repair	(16, 34, 53, 94–107)

## 5 Why MIRI is essentially a spatiotemporally hierarchical immunometabolic disease

MIRI is not a pathological event resulting from the mechanical superposition of metabolic abnormalities occurring simultaneously in multiple cells, but rather an immunometabolic process that continuously reorganizes over time and space. The initial minutes to hours following reperfusion determine whether the injury is rapidly amplified; the subsequent days determine whether inflammation can be orderly resolved and transition into repair. At the same time, different regions within the heart do not share the same microenvironment: the core, border, and distal myocardium exhibit significant differences in perfusion status, cell death burden, metabolite concentrations, mechanical stress, and immune cell composition. For this reason, MIRI is better understood as a spatiotemporally stratified immunometabolic disease: the same metabolic molecule, the same type of immune cell, or even the same signaling pathway may perform entirely opposite functions across different time windows and tissue sites.

### 5.1 Metabolic switching from acute injury amplification to inflammatory resolution

The temporal dimension of MIRI is best understood as a staged shift in metabolic control rather than as a simple progression from inflammation to repair (110). During the earliest phase of reperfusion, the local microenvironment is dominated by hypoxia, reactive oxygen species (ROS) burst, succinate and lactate accumulation, release of damage-associated molecular patterns, and a high burden of dying cells. This setting rapidly favors glycolytic and redox-intensive programs in infiltrating myeloid cells. Importantly, recent single-cell studies indicate that even this early inflammatory phase is not metabolically uniform. Neutrophils respond first, but they do so through heterogeneous states rather than through a single pro-damaging program (125). Cardiac immune single-cell profiling has identified temporally distinct neutrophil populations, including Ccl3^hi and Ym-1^hi subsets, with Ym-1^hi neutrophils emerging early and showing cardioprotective properties, at least in part through effects on macrophage polarization (98). In parallel, a distinct S100a9^hi monocyte-derived macrophage subset enters the heart within hours after reperfusion and amplifies acute inflammation through a MyD88/NF-κB/NLRP3 axis (81). Thus, the early phase of MIRI is defined not simply by inflammatory cell influx, but by the rapid dominance of specific glycolytic and inflammasome-linked immune states that translate metabolic stress into tissue injury.

As reperfusion progresses, metabolic control does not automatically revert to homeostasis. Instead, the tissue enters a vulnerable transition window in which inflammatory amplification may either persist or be redirected toward resolution. In this phase, recovery depends less on the passive disappearance of inflammatory signals than on the coordinated re-establishment of oxidative and efferocytic programs. Macrophages that shift toward OXPHOS/FAO-supported states, enhance efferocytosis, and engage buffering pathways such as itaconate-related programs are more likely to support apoptotic cell clearance, limit inflammatory persistence, and promote repair. Recent studies further indicate that this transition is actively regulated rather than passively acquired. Resident macrophage HIF-1α-dependent monocyte fate control and Maresin 1–driven efferocytosis illustrate how inflammatory resolution requires coordinated recovery of oxidative and pro-resolving programs, rather than passive decay of inflammatory signals (90, 115).

**Figure 2:**
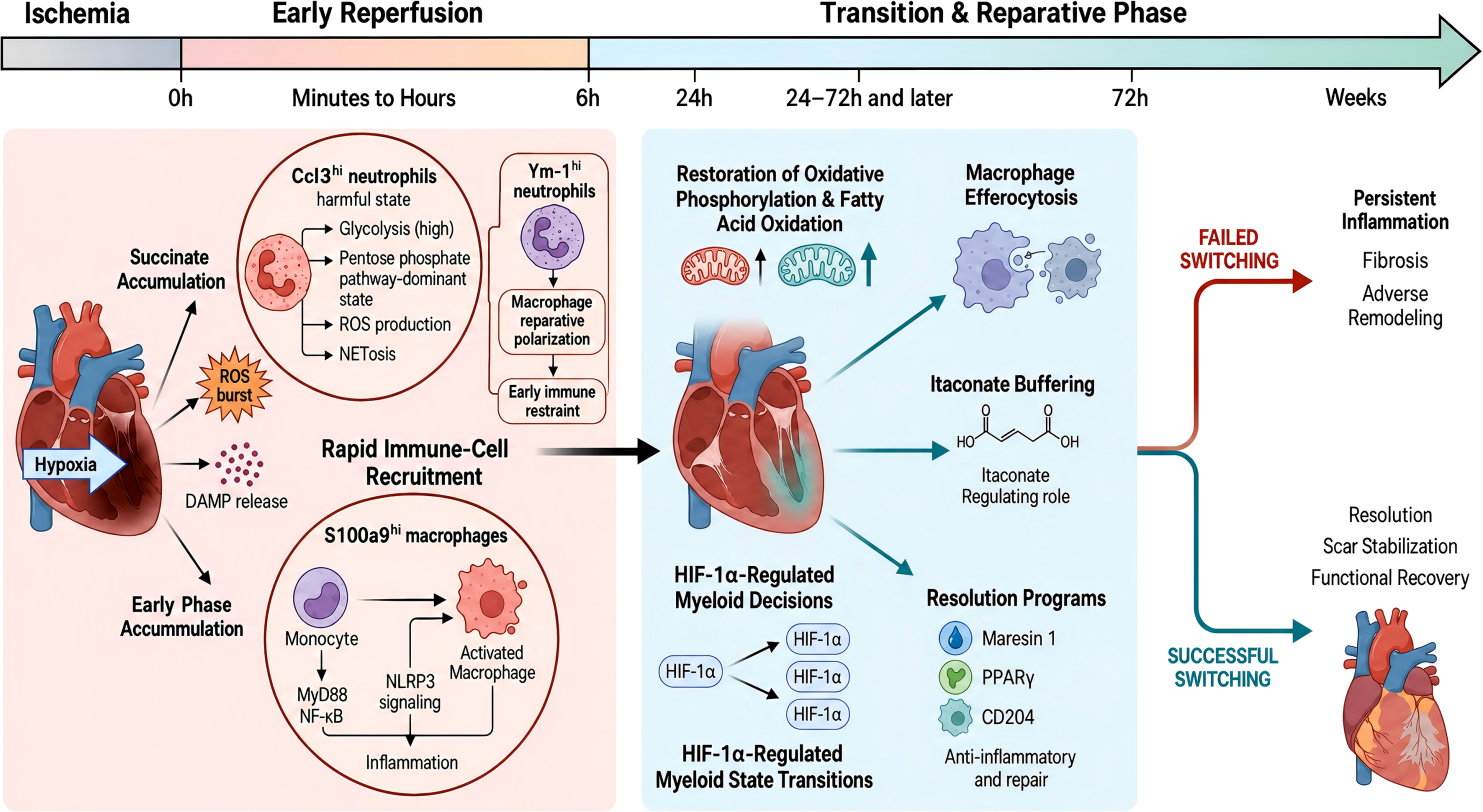
Temporal switching of immunometabolic programs during MIRI. The temporal progression of MIRI is defined by staged changes in metabolic control. During the earliest minutes to hours of reperfusion, hypoxia, succinate accumulation, lactate accumulation, reactive oxygen species burst, damage-associated molecular pattern release, and cell debris favor glycolytic and redox-intensive immune states. Ccl3^hi neutrophils and S100a9^hi monocyte-derived macrophages promote acute inflammatory amplification through ROS production, NETosis, and MyD88/NF-κB/NLRP3-related signaling, whereas Ym-1^hi neutrophils may contribute to early immune restraint by supporting reparative macrophage polarization. During the transition and reparative phases, successful recovery depends on restoration of oxidative phosphorylation, fatty acid oxidation, macrophage efferocytosis, itaconate-associated buffering, and Maresin 1–PPARγ–CD204-linked resolution programs. Failure of this metabolic switch promotes persistent inflammation, fibrosis, and adverse remodeling.

Accordingly, the temporal logic of MIRI lies not in the mere presence or absence of inflammation, but in whether metabolic control can shift, within the appropriate time window, from acute injury amplification toward controlled resolution. Failure of this switch prolongs glycolytic and inflammasome-linked myeloid persistence, sustains inflammatory crosstalk with stromal cells, and promotes adverse remodeling. By contrast, successful transition to oxidative, efferocytic, and resolution-biased states supports scar stabilization and functional recovery. In this sense, the temporal dimension of MIRI is best viewed as a sequence of competing metabolic programs whose balance, rather than their absolute presence, determines the trajectory from acute injury to repair (**Figure 2**).

### 5.2 Infarct core, border zone, and remote myocardium as distinct metabolic niches

The reperfused heart in MIRI should be viewed as a spatially organized system of distinct metabolic–immune niches rather than as a single ischemic lesion (57, 126). At minimum, these niches include the infarct core, the border zone, and the remote myocardium, each defined by a different combination of perfusion status, cell death burden, immune infiltration, and metabolic stress (57, 126). The infarct core is typically the region of poorest perfusion and greatest necrotic load. Accordingly, it accumulates reactive oxygen species, damage-associated molecular patterns, and metabolically stressed inflammatory cells. Recent spatial transcriptomic analyses of myocardial ischemia-reperfusion have shown that infarct cores are preferentially enriched for ferroptosis- and mitophagy-related programs, whereas incomplete infarct regions are enriched for neutrophil-, MAPK-, and IL-17-related signaling (57). Integration with single-cell data further revealed immune infiltration within infarcted tissue and dynamic activation of Piezo1-associated Ca^2+ dysregulation in specific cardiomyocyte subclusters (57). Together, these findings indicate that the reperfused lesion is internally compartmentalized and that spatial heterogeneity reflects coordinated variation in both cellular composition and metabolic signaling.

Among these niches, the border zone is the most dynamically plastic (126). It is not merely an anatomical extension of the infarct core, but a transcriptionally and mechanically distinct interface between severely injured and relatively preserved myocardium. Single-cell and spatial transcriptomic studies have shown that the border zone forms early after myocardial injury and contains spatially restricted cardiomyocyte states, including BZ1 (Nppa^+ Xirp2^−) and BZ2 (Nppa^+ Xirp2^+), that differ from both infarct and remote regions (126). In parallel, mechano-sensing programs, including Csrp3-associated transcriptional responses, are enriched in this region and influence subsequent ventricular remodeling. In MIRI, these observations suggest that the border zone is a metabolically and mechanically destabilized interface in which lactate, succinate, debris-derived inflammatory signals, and altered force transmission converge to shape cell-state transitions. This may explain why the border zone is particularly important in determining whether local programs remain locked in injury amplification or shift toward adaptive reconstruction.

**Figure 3:**
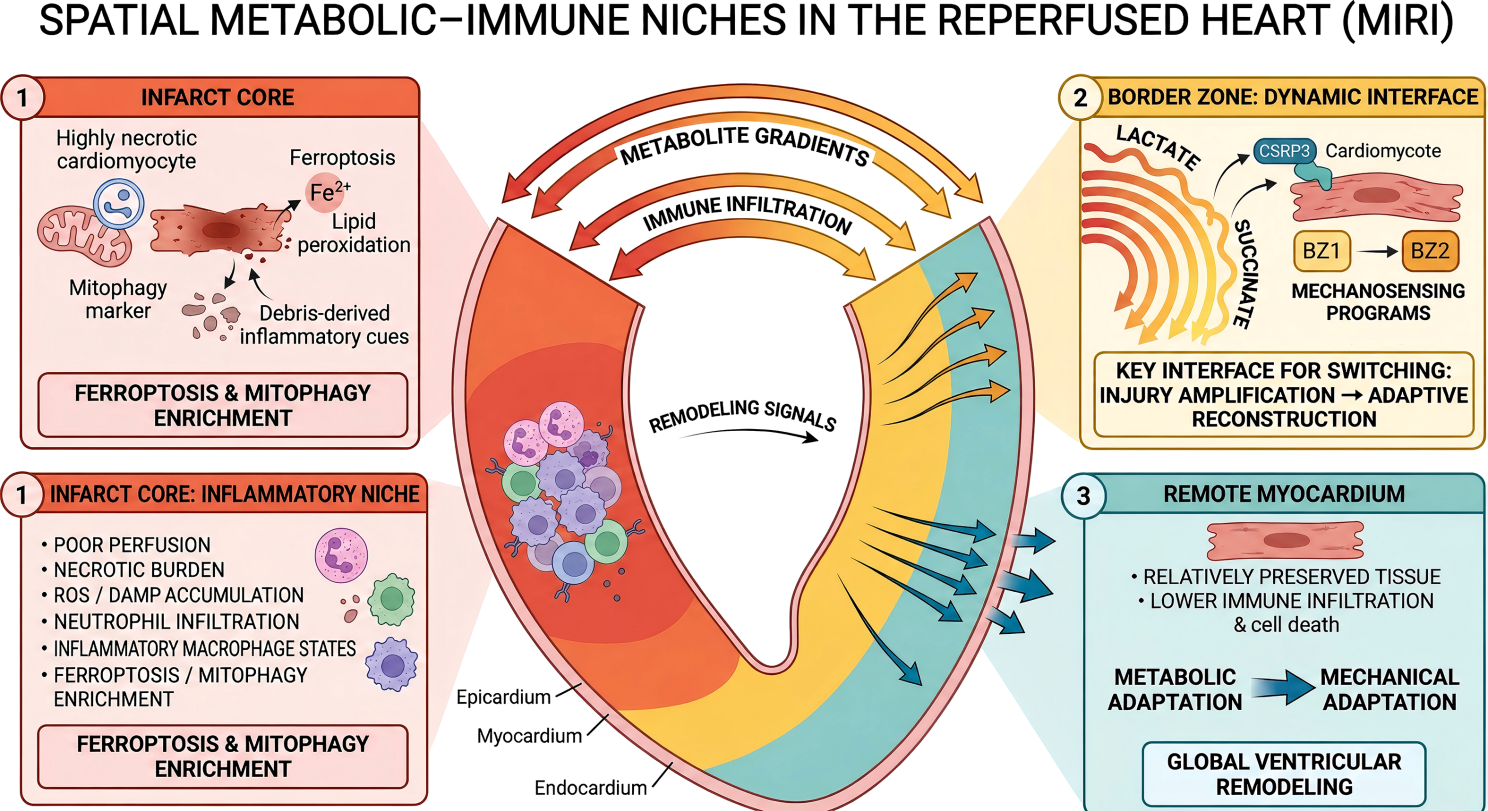
Spatial metabolic–immune niches in the reperfused heart during MIRI. The reperfused myocardium is spatially organized into distinct metabolic–immune niches rather than a uniform ischemic lesion. The infarct core is characterized by severe perfusion loss, high necrotic burden, mitochondrial stress, ferroptosis, mitophagy-related programs, debris-derived inflammatory cues, and immune-cell accumulation. The border zone is a dynamic interface between severely injured and relatively preserved myocardium. In this region, lactate and succinate gradients, altered mechanical force transmission, spatially restricted cardiomyocyte states, and mechano-sensing programs such as Csrp3-associated responses converge to shape cell-state transitions. The remote myocardium retains a more preserved tissue environment with lower immune infiltration and overt cell death, but contributes to global ventricular remodeling through metabolic and mechanical adaptation. These regional niches determine whether local programs remain injury-amplifying or shift toward adaptive reconstruction.

By contrast, the remote myocardium maintains a microenvironment that is closer to baseline homeostasis, with lower levels of immune infiltration and overt cell death. Nevertheless, it should not be regarded as biologically inert. Rather, the remote region provides the metabolic and mechanical context within which global ventricular remodeling evolves (127). The spatial organization of MIRI is therefore hierarchical rather than binary: the infarct core represents the zone of maximal metabolic collapse, the border zone functions as the principal interface of state transition and remodeling plasticity, and the remote myocardium contributes to longer-range adaptive or maladaptive remodeling. Accordingly, the spatial dimension of MIRI should be interpreted not simply as anatomical subdivision, but as the organization of distinct metabolic niches that differentially shape immune behavior, tissue injury, and repair outcome (**Figure 3**).

## 6 The therapeutic potential and clinical translation pathways of MIRI

Inflammatory responses following myocardial ischemia-reperfusion injury are tightly coupled to metabolic imbalance; therefore, immunometabolic targeting has become an attractive strategy for limiting acute injury and promoting repair. The central translational challenge is not the absence of candidate targets, but the difficulty of matching the correct target to the correct time window, cell state, and lesion site. Strategies with realistic clinical potential should therefore move beyond broad anti-inflammatory, antioxidant, or energetic support and instead test whether a defined metabolic program can be safely redirected in a selected patient and tissue context.

### 6.1 Timing is more critical than a single target

The temporal hierarchy discussed above has direct therapeutic implications. Interventions applied during the perireperfusion window are most likely to affect acute injury amplification, including glycolytic-redox stress, mitochondrial oxidant burst, and inflammasome activation. By contrast, interventions applied during the early remodeling phase should prioritize mitochondrial recovery, efferocytosis, oxidative metabolism, and resolution programs. Thus, metabolic nodes such as HIF-1α, succinate, AMPK, and mTOR should not be classified as uniformly protective or harmful. Their therapeutic significance depends on the phase in which they are targeted.

This principle is increasingly reflected in clinical trial design. In the EMMY trial, empagliflozin was initiated within 72 hours after percutaneous coronary intervention (PCI) and was associated with a greater reduction in N-terminal pro-B-type natriuretic peptide (NT-proBNP), together with favorable changes in ventricular structure and function (128). By contrast, in EMPACT-MI, empagliflozin was started within 14 days after acute myocardial infarction; the primary composite endpoint was not significantly reduced, although first and total heart failure hospitalizations were lower in exploratory analyses (129). These findings are not necessarily contradictory. Rather, they suggest that the closer an intervention is aligned with the perireperfusion window and the onset of early remodeling, the more likely it is to capture effects that are directly relevant to MIRI itself. The EMPA-PCI trial is designed to extend this logic by moving treatment to the period around PCI and by using no-reflow and MRI-defined infarct size as endpoints (130). Compared with traditional long-term post-discharge follow-up, this design is more closely matched to the temporal pathology of MIRI.

### 6.2 From broad-spectrum immunosuppression to state-specific reprogramming

The immune-cell heterogeneity discussed above has direct therapeutic implications. Rather than suppressing whole lineages, future interventions should target pathogenic states within defined time windows (81, 98). For neutrophils, this means avoiding indiscriminate depletion that may remove protective subsets as well as damaging ones (81, 98, 103). For macrophages, it means redirecting inflammatory glycolytic states toward oxidative, efferocytic, or pro-resolving programs (81, 103).

Within this framework, the significance of metabolic nodes must also be reinterpreted. Their value lies not merely in whether they are classified as inflammatory or metabolic mediators, but in whether they control transitions between pathogenic and resolving immune states. PGC-1α provides a representative example. PGC-1α was shown to suppress pro-inflammatory macrophage polarization and mitigate myocardial ischemia-reperfusion injury through dual regulation of SUCLG1/succinate metabolism and TRAF5 expression, thereby linking oxidative metabolic recovery directly to restraint of TLR4/NF-κB-driven inflammatory programming (104). Similarly, the therapeutic relevance of S100A8/A9-related signaling, lactate-associated epigenetic remodeling, and itaconate derivatives lies in their ability to shift immune cells away from injury-amplifying states and toward pro-resolving or reparative trajectories (131, 132). Accordingly, future immunometabolic therapy in MIRI is more likely to benefit from selective reprogramming of defined immune states than from nonspecific suppression of entire cell populations.

### 6.3 From systemic administration to site- and cell-specific enrichment

A major limitation of immunometabolic therapy in MIRI is that correct target selection does not guarantee effective intervention unless the agent reaches the relevant tissue compartment and cellular state. For this reason, site-directed and cell-specific delivery strategies have become increasingly important. Mitochondrial targeting illustrates this shift (133). In diabetic settings, MitoQ has been shown to attenuate myocardial ischemia-reperfusion injury by enhancing PINK1/Parkin-dependent mitophagy, suggesting that restoration of mitochondrial quality control may protect not only cardiomyocytes but also the local immune microenvironment by limiting mtROS accumulation and inflammatory signal propagation (133, 134). More recently, biomimetic nanodelivery platforms have begun to combine lesion enrichment with selective modulation of immune pathways. Macrophage-membrane-coated S100A9-siRNA nanoparticles accumulate in inflamed myocardium, reduce S100A8/A9 signaling, and improve infarct size and cardiac function (135). Likewise, engineered neutrophil-membrane-based nanosystems exploit the LFA-1/ICAM-1 axis for inflammation-responsive targeting and, through delivery of S100A9 siRNA or inhibition of integrin α9, reduce neutrophil infiltration, NET formation, and microthrombus burden (135).

The significance of these approaches lies not simply in improving drug accumulation, but in redefining the therapeutic logic of MIRI. The goal is no longer only to identify a protective molecule, but to deliver it to the appropriate lesion site, cell population, and subcellular context. This principle is illustrated by hierarchical delivery systems that use neutrophil-derived NET-associated networks to direct therapeutic cargo first to injured myocardium and subsequently to cardiomyocyte subcellular organelles, thereby restoring SERCA function and improving remodeling (136). Although these strategies remain far from routine clinical application, they establish a clear translational direction: future precision therapy for MIRI is likely to depend less on systemic administration of single-pathway drugs and more on the integration of metabolic targeting, spatial enrichment, and cell-specific recognition.

### 6.4 Drug repurposing, mitochondrial targeting, and intervention at metabolic nodes

Among current translational strategies for MIRI, drug repurposing remains the closest to clinical application, whereas mitochondrial targeting and metabolic-node intervention represent the most conceptually aligned preclinical directions. Metformin illustrates this distinction well. Preclinical studies continue to support its cardioprotective effects through AMPK activation, suppression of NOX4, and modulation of the AMPK–HMGCR–ROS axis (122, 137). More recent work has also linked metformin to reduced PANoptosis and oxidative stress in experimental MIRI (138). However, the clinical evidence remains indirect, because available signals derive largely from acute myocardial infarction or cardiometabolic-risk settings rather than from trials designed specifically to test acute anti-MIRI efficacy. Accordingly, metformin is best described as a mechanistically plausible and clinically accessible candidate, rather than as an established anti-MIRI therapy.

The translational case for sodium-glucose cotransporter 2 (SGLT2) inhibitors is more closely related to the peri-reperfusion setting. In EMMY, empagliflozin initiated within 72 hours after PCI was associated with a greater decline in NT-proBNP and favorable changes in ventricular structure and function (128). By contrast, in EMPACT-MI, empagliflozin started within 14 days after acute myocardial infarction did not significantly reduce the primary composite endpoint, although first and total heart failure hospitalizations were lower in secondary or exploratory analyses (129, 139). EMPA-PCI is designed to move empagliflozin even closer to the reperfusion event, including pre-PCI administration and endpoints such as no-reflow and MRI-defined infarct size. Taken together, these studies suggest that SGLT2 inhibitors are best regarded as clinically tractable candidates with substantial translational potential, while their direct anti-MIRI effects still require confirmation in trials more tightly aligned with the peri-reperfusion window and with MIRI-specific endpoints.

By contrast, interventions at specific metabolic nodes remain largely preclinical, but they may better reflect the logic of the immune–cardiac metabolic circuit. 4-octyl itaconate (4-OI) has been shown to attenuate myocardial ischemia-reperfusion injury while promoting angiogenesis, indicating that it can bias the injured microenvironment toward repair rather than merely suppress inflammation (105). Similarly, an acid-triggered peptide–drug conjugate that combines 4-octyl itaconate with the mitochondria-targeting peptide SS31 (elamipretide) has been developed to restore redox homeostasis and improve mitochondrial function in experimental myocardial I/R injury (140). The value of these strategies lies in the fact that they do not simply intensify antioxidant therapy. Instead, they attempt to intervene simultaneously in metabolic buffering, inflammatory restraint, and tissue reconstruction. For this reason, future precision therapy for MIRI is likely to depend less on single-system drug administration and more on coordinated combinations of drug repurposing, mitochondrial targeting, and state-relevant metabolic-node modulation.

### 6.5 Practical translational priorities

Three priorities emerge from the therapeutic literature. First, trials should align intervention timing with MIRI biology. Peri-reperfusion and early remodeling windows should be tested with MIRI-specific endpoints such as no-reflow, cardiac magnetic resonance-defined infarct size, microvascular obstruction, myocardial salvage, and early biomarker trajectories, rather than relying only on late heart-failure outcomes (128–130). Second, patient enrichment should be incorporated. Individuals with large area at risk, microvascular obstruction, reduced left ventricular ejection fraction, diabetes, advanced age, or high inflammatory/metabolic biomarkers are more likely to reveal whether an immunometabolic intervention modifies injury rather than simply improving background cardiovascular risk. Third, target selection should be linked to measurable cell states or sensors, such as S100A8/A9-related myeloid activation, lactylation-associated neutrophil trafficking, purinergic enzyme activity, or mitochondrial quality-control markers. These priorities do not establish any single therapy as definitive, but they provide a more testable path from mechanistic discovery to clinical translation.

## 7 From descriptive omics to mechanistic profiling

**Figure 4:**
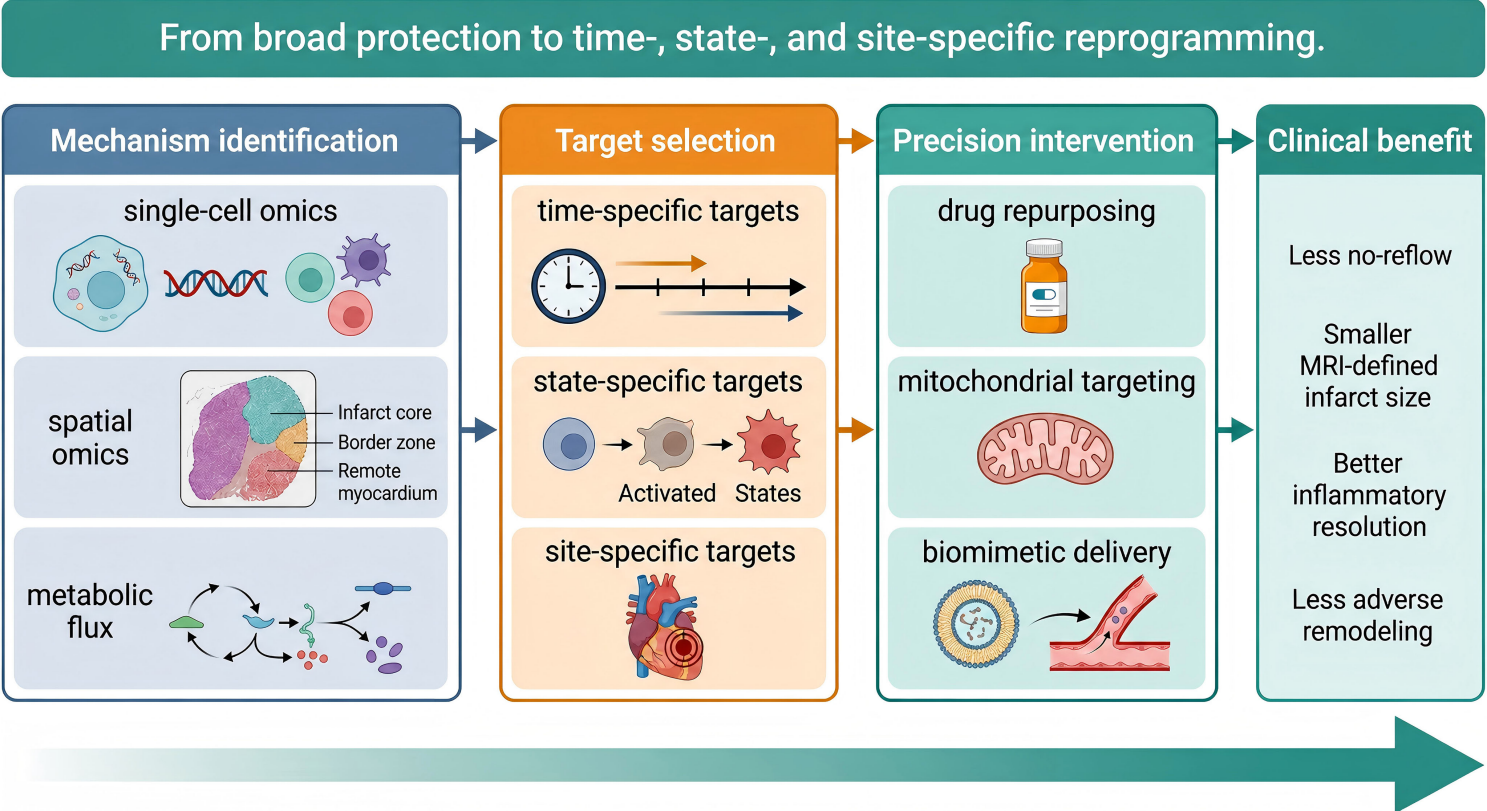
Precision immunometabolic therapy in MIRI: from mechanism identification to targeted intervention. Precision immunometabolic therapy for MIRI requires integration of mechanism identification, target selection, and spatially controlled intervention. High-resolution approaches, including single-cell omics, spatial omics, and metabolic flux analysis, can identify pathogenic immune-cell states, lesion-specific metabolic niches, and functionally relevant substrate-use programs. These data support the prioritization of time-specific, state-specific, and site-specific therapeutic targets. Candidate interventions include drug repurposing, mitochondrial targeting, and biomimetic delivery strategies designed to enrich therapeutic agents in the appropriate lesion region, cell population, or subcellular compartment. The goal is to move beyond broad-spectrum cardioprotection toward selective immunometabolic reprogramming, with potential benefits including reduced no-reflow, smaller MRI-defined infarct size, improved inflammatory resolution, and less adverse remodeling.

Immune-metabolic reprogramming in MIRI is temporally dynamic, spatially heterogeneous, and highly dependent on cell-state. For this reason, conventional bulk transcriptomic, proteomic, and metabolomic analyses can identify average molecular changes, but they are poorly suited to resolve the questions that now matter most. These include which cell populations enter pro-inflammatory or pro-reparative metabolic states after reperfusion, when these transitions occur, where they are localized within the infarct core, border zone, or remote myocardium, and whether such changes are merely associated with disease progression or actively drive it. The central limitation of the field has therefore not been a lack of data, but a lack of analytical frameworks capable of integrating time, space, cell-state, and actual metabolic activity. Against this background, the importance of emerging technologies lies not in producing additional descriptive maps, but in enabling the immune–cardiac metabolic circuit of MIRI to be resolved into identifiable, spatially localized, and functionally testable mechanistic networks (**Figure 4**).

### 7.1 Single-cell omics

Single-cell and single-nucleus omics have reshaped the study of MIRI not primarily by expanding the list of cell types present, but by revealing that each major immune compartment contains multiple, temporally and functionally distinct states. Earlier models often treated neutrophils, macrophages, and lymphocytes as relatively homogeneous populations and classified them broadly as pro-inflammatory or reparative. By contrast, recent single-cell studies indicate that disease progression and repair are more closely linked to specific cell states and state trajectories than to whole cell classes. In neutrophils, certain subsets are associated with oxidant production and amplified NET formation, whereas others appear more consistent with inflammation resolution (98). In macrophages, the traditional M1/M2 framework is increasingly being replaced by a continuum that spans glycolytic inflammatory states, phagocytic states, lipid-associated programs, and reparative phenotypes (16, 81). A similar principle is emerging in lymphocytes, where effector T cells, Tregs, and regulatory B-cell populations appear to operate with different metabolic dependencies and functional thresholds under distinct microenvironmental conditions.

Accordingly, the major contribution of single-cell omics to MIRI immunometabolism is a shift from cell-type cataloguing to cell-state and trajectory analysis. This conceptual shift provides the cellular resolution needed for state-specific therapeutic thinking, as discussed in Section 6.2. However, single-cell omics alone cannot establish metabolic function, because most current studies infer glycolysis, fatty acid oxidation, or mitochondrial activity from transcriptional signatures rather than directly measuring metabolite abundance or flux. Its full mechanistic value in MIRI will therefore depend on integration with spatial profiling and orthogonal metabolic validation.

### 7.2 Spatial transcriptomics and spatial metabolomics

Spatial omics adds a critical dimension to MIRI research by identifying where distinct cellular and metabolic states arise within injured tissue. This is particularly important because the reperfused heart is not a homogeneous lesion, but a spatially stratified system composed of infarct core, border zone, and remote myocardium (57, 141). These regions differ substantially in oxygen availability, substrate utilization, cell death burden, mechanical stress, and immune-cell composition. Consequently, the same metabolic program may have very different biological implications depending on its location. Enhanced glycolysis in neutrophils within the infarct core is more likely to reflect acute inflammatory amplification, whereas oxidative metabolic programs in macrophages or endothelial cells within the border zone may indicate transition toward repair (57, 141).

Within this framework, the value of spatial transcriptomics lies in its ability to place cell states back into tissue architecture. Its contribution is not limited to validating the localization of single-cell findings. Rather, it helps explain why the border zone functions as a critical interface between injury amplification and tissue reconstruction, why identical signaling pathways may carry different meanings in the core and peripheral regions, and why resident and recruited cells can follow distinct metabolic programs within the same organ (57, 141). In this way, spatial transcriptomics moves the immune–cardiac metabolic circuit from a conceptual model to a histologically resolved framework.

Spatial metabolomics extends this logic by converting inferred metabolic programs into directly observable molecular gradients (141). Its particular value lies in revealing the distribution of lactate, lipid intermediates, energy metabolites, and oxidative stress-related molecules across the core, border, and remote regions (141). This provides more direct evidence for how the local metabolic milieu shapes immune-cell recruitment, polarization, and fate. In MIRI, the importance of spatial metabolomics therefore lies not in generating additional descriptive maps, but in showing that immune-cell infiltration and functional polarization occur within a tissue field that has already been organized by metabolite gradients. Taken together, spatial transcriptomics and spatial metabolomics provide a key framework for understanding the spatial logic of immunometabolic heterogeneity in MIRI.

### 7.3 Metabolic flux and functional perturbation

A major limitation in current MIRI immunometabolic research is that most conclusions remain based on transcriptional inference rather than direct measurement of substrate use, metabolic flux, and functional consequence. Although single-cell and spatial omics have substantially improved resolution, expression of glycolytic or fatty acid oxidation–related genes does not necessarily indicate that these pathways are functionally dominant. This limitation is particularly important in MIRI, where metabolic states change rapidly across time, space, and cell type. As a result, transcription-based analyses alone may overestimate, misclassify, or oversimplify the true metabolic behavior of injured cardiac and immune cells.

For this reason, metabolic flux analysis and functional perturbation are essential for moving the field from descriptive profiling to mechanistic interpretation. Isotope tracing, substrate tracing, and direct flux measurements can determine which substrates are actually used, at what rate, and through which pathways (142, 143). Functional perturbation approaches, including pharmacologic inhibition, gene editing, conditional cell-specific deletion, and state-selective intervention, are then required to test whether a given metabolic node is merely associated with disease progression or actively drives it. This distinction is especially important in MIRI, because many metabolic pathways show context-dependent or even opposing effects across different stages and tissue regions. Only by combining state identification with causal testing can one determine whether a given node represents a therapeutic target or simply a marker of lesion evolution.

Accordingly, metabolic flux analysis and functional perturbation should not be regarded as secondary supplements to single-cell and spatial omics. Rather, they are the critical step that converts high-resolution observation into mechanistic understanding. Future progress in MIRI immunometabolism will therefore depend not only on identifying new subpopulations or spatial niches, but also on answering more decisive questions: Does a given metabolic program actively drive injury amplification? Do local metabolic gradients shape cell fate and function? And, if these pathways are selectively manipulated, can cardiac outcome be altered in a predictable manner?

## 8 Discussion

The central conclusion of this review is that MIRI is best interpreted as a dysregulated immune–cardiac metabolic communication disorder rather than as a set of parallel cell-intrinsic injuries. The evidence reviewed here connects three processes that are often discussed separately: metabolic signal release from injured resident cardiac cells, metabolic decoding by infiltrating immune populations, and spatiotemporally restricted transitions between injury amplification and repair. This framework does not replace established mechanisms such as ROS burst, calcium overload, microvascular dysfunction, or regulated cell death. Instead, it places them within a circuit in which the biological meaning of a pathway depends on cell type, tissue location, and timing.

Several unresolved issues emerge from this circuit-based view. First, many metabolites cannot be classified as uniformly protective or pathogenic. Lactate may support early cardiomyocyte adaptation under selected conditions, but excessive glycolysis-derived lactate can promote lactylation-dependent ferroptosis, pyroptosis, or neutrophil trafficking. Succinate can amplify mitochondrial ROS generation intracellularly, whereas extracellular succinate may serve as an inflammatory cue whose effects depend on efflux, retention, and receptor context. Second, how metabolic information is transferred within spatially restricted niches remains insufficiently resolved. Myeloid–stromal crosstalk, exemplified by S100a9^hi macrophage-associated fibrotic remodeling, suggests that inflammatory and metabolic programs can be redistributed from immune cells to fibroblasts and other stromal compartments. However, the spatial handoff of lactate, succinate, ATP/adenosine, lipid mediators, and mitochondrial danger signals within the mechanically destabilized border zone remains incompletely mapped.

Methodologically, the field still relies heavily on transcriptional inference, whereas direct measurements of metabolite abundance, substrate use, and metabolic flux remain limited. This limitation is particularly important because the same transcriptional program may correspond to different metabolic activity depending on oxygen availability, substrate access, tissue location, and cell state. Future studies should therefore pair single-cell and spatial profiling with isotope tracing, spatial metabolomics, and cell-state-specific perturbation. In parallel, translation will require models that better reflect human myocardial infarction, including aging, diabetes, variable reperfusion timing, and standard-of-care pharmacotherapies.

Several mechanisms discussed in this review remain biologically unresolved. Lactate can support early cardiomyocyte adaptation through HSPA12A-dependent maintenance of aerobic glycolysis and histone lactylation, but excessive glycolysis-derived lactate may promote lactylation-dependent ferroptosis, pyroptosis, or neutrophil trafficking (34, 37, 47–49, 184). Succinate can amplify intracellular mitochondrial ROS generation, yet extracellular succinate may also serve as an inflammatory cue whose effects depend on efflux, retention, and receptor context (45, 115, 116). HIF-1α can reinforce inflammatory glycolysis in some myeloid states, while resident macrophage HIF-1α controls monocyte fate specification in a manner that may support repair (114). Similarly, neutrophils and macrophages contain both injury-amplifying and repair-associated states, making broad depletion conceptually problematic (81, 98, 103, 125). These examples underscore that many immunometabolic nodes are not intrinsically protective or pathogenic; their function depends on dose, compartment, cell state, and timing.

The current literature also has important limitations. First, most single-cell and spatial studies infer metabolism from gene-expression signatures rather than directly measuring metabolite abundance, substrate use, or flux. Second, many mechanistic studies rely on mouse models with controlled ischemic duration, age, diet, and medication exposure, whereas human myocardial infarction occurs in patients with heterogeneous comorbidities, aging-related immune changes, variable reperfusion timing, and standard-of-care therapies that reshape baseline metabolism. Third, several attractive targets, including lactate sensing, SUCNR1-dependent succinate signaling, itaconate derivatives, and lymphocyte metabolic programs, remain supported by incomplete cell-specific or flux-based evidence in MIRI. Finally, some studies use permanent infarction rather than ischemia-reperfusion models; these data are informative for repair biology but should not be treated as direct proof for acute reperfusion injury.

Overall, the aim of this review is not merely to restate that immunometabolism is relevant to MIRI. Rather, it proposes that the field should move from descriptive mechanism mapping toward testable circuit biology. In such a framework, the injured heart is viewed as a source of metabolic signals, immune cells as state-dependent decoders, and the reperfused lesion as a spatiotemporally stratified system. This perspective supports a more precise experimental and therapeutic logic: define the cell state, locate the niche, measure the flux, perturb the node, and test whether cardiac outcome changes in a predictable manner.
